# Investigation of Genetic Susceptibility to Blastomycosis Reveals Interleukin-6 as a Potential Susceptibility Locus

**DOI:** 10.1128/mBio.01224-19

**Published:** 2019-06-18

**Authors:** Richard M. Merkhofer, Mary B. O’Neill, Donny Xiong, Nydiaris Hernandez-Santos, Hannah Dobson, J. Scott Fites, Abigail C. Shockey, Marcel Wuethrich, Caitlin S. Pepperell, Bruce S. Klein

**Affiliations:** a Department of Pediatrics, School of Medicine and Public Health, University of Wisconsin—Madison, Madison, Wisconsin, USA; b Laboratory of Genetics, University of Wisconsin—Madison, Madison, Wisconsin, USA; c Department of Medical Microbiology and Immunology, School of Medicine and Public Health, University of Wisconsin—Madison, Madison, Wisconsin, USA; d Department of Medicine, School of Medicine and Public Health, University of Wisconsin—Madison, Madison, Wisconsin, USA; Duke University; NIH; University of Arizona

**Keywords:** *Blastomyces*, cell-mediated immunity, immunogenetics

## Abstract

Genetic differences are hypothesized to underlie ethnic disparities in incidence rates of the endemic systemic mycoses, including blastomycosis. Individuals of Hmong ancestry display elevated risk for this serious fungal infection. Here, we interrogated the genomes of Wisconsin (WI) Hmong blastomycosis patients using homozygosity mapping to uncover regions of the genome that are likely shared among the greater Hmong population and filtered for variants with high potential to affect disease susceptibility. This approach uncovered 113 candidate susceptibility variants, and among the most promising are those in genes involved in the interleukin-17 (IL-17) response. In particular, we identified 25 linked variants near the gene encoding IL-6 (*IL6*). We validated differences in cytokine production between Hmong and European volunteers and formally demonstrated a critical role for IL-6 in the development of adaptive immunity to Blastomyces dermatitidis. Our findings suggest that the dysregulation of IL-17 responses underlies a recently reported and poorly understood ethnic health disparity.

## Abstract (precis)

Blastomycosis is a potentially life-threatening infection caused by the fungus Blastomyces dermatitidis. As with related fungal diseases, blastomycosis is noted to affect some populations more than others. These patterns of illness are often not related to predisposing conditions or exposure risks; thus, genetic differences are thought to underlie these health disparities. People of Hmong ancestry in Wisconsin are at elevated risk of blastomycosis compared to the general population. We studied the genetic codes of Hmong blastomycosis patients and identified candidate sites in their genomes that may explain their susceptibility to this infection. We further studied one particular region of the genome that is involved with the immune processes that fight B. dermatitidis. Our work revealed population differences in the response to fungi. A better understanding of the genetic underpinnings of susceptibility to infectious diseases has broader implications for community health, especially in the paradigm of personalized medicine.

## INTRODUCTION

Persons of Hmong ancestry in Wisconsin (WI) have a strikingly elevated incidence of blastomycosis relative to those of European ancestry (168 versus 13 per 100,000, respectively), and this disparity is not attributable to differential exposure to the pathogen or comorbidities that alter susceptibility (1). This finding is consistent with previous reports that endemic fungal diseases (e.g., blastomycosis, histoplasmosis, and coccidioidomycosis) affect some populations with higher rates or severity (2). Therefore, it has been widely hypothesized that human genetic variation is an important determinant of the outcome of exposure to dimorphic fungal pathogens. To date, few genetic polymorphisms associated with susceptibility to dimorphic fungal infection have been reported, and variants that confer risk of infection are understood to be major mutations of Mendelian loci. Reports of such variants account for only a few individual patient’s cases (3, 4).

The few genetic studies of Hmong populations have revealed that they are highly distinct from those of other East Asian populations. Overall homozygosity is high in the Hmong (5, 6), consistent with historically high rates of within-group marriage and recent population bottlenecks due to repeated migrations, geographic dispersal, and population losses from military conflict (7). This history is expected to lead to increased levels of autozygosity, that is, homozygosity in which two alleles are identical by descent. We hypothesize that homozygous regulatory variant(s) common to the WI Hmong affect their immune responses to fungi, leading to an elevated incidence of blastomycosis.

In this report, we used a bioinformatic strategy to identify polymorphisms that may account for the increased risk of blastomycosis among the WI Hmong and functionally validated our findings with *in vitro* studies of human cells and a murine model of blastomycosis. Briefly, we used a disease mapping strategy designed to exploit the high levels of autozygosity in the study population, as has been used previously to identify homozygous alleles associated with various diseases (8). We then leveraged human genetic resources characterizing variants across the genome to prioritize variants that may mediate susceptibility to infection. We uncovered a set of 25 polymorphisms near the gene encoding interleukin-6 (IL-6), *IL6*. We focused on these variants in part due to the role of IL-6 in nurturing IL-17 responses, which are protective in murine models of blastomycosis (9). We report significant differences in cytokine production between populations, with Hmong donors producing less IL-6 than do their European counterparts and also less IL-17 by memory CD4^+^ T cells in response to fungi. Finally, we formally demonstrate the importance of IL-6 in the development of adaptive immunity and resistance in a murine model of pulmonary infection with Blastomyces dermatitidis.

## RESULTS AND DISCUSSION

### Analysis of Hmong blastomycosis patient genomes reveals 113 candidate susceptibility variants.

**FIG 1:**
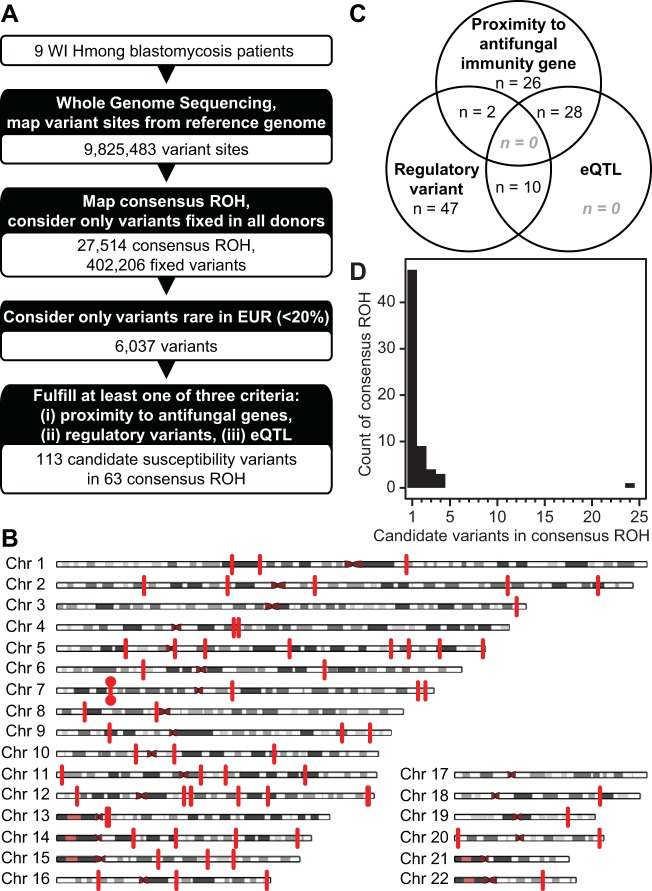
Analysis of Hmong blastomycosis patient genomes reveals candidate susceptibility variants. Whole-genome sequencing was performed for nine blastomycosis patients of Hmong ancestry. (A) Autozygosity mapping was performed, and variants within consensus runs of homozygosity (ROH) were subjected to a series of filters, leading to the identification of candidate susceptibility variants. Filters included that variants must be fixed in all Hmong donors, be rare in European populations, and meet at least one of three criteria to identify variants with a higher likelihood of impacting antifungal immunity. (B) Distribution of ROH harboring candidate susceptibility variants (red vertical lines) on autosomes. ROH at the *IL6* locus on chromosome (Chr) 7 are marked with a red dot; for details of the *IL6* locus, see Fig. 2. (C) Number of variants meeting individual or multiple criteria. (D) Frequency of candidate variants identified in consensus ROH; the consensus ROH overlapping *IL6* harbors the largest number of candidate variants (*n *=* *24). EUR, Europe.

To identify variants that may affect susceptibility to infection, we performed whole-genome sequencing (WGS) on 11 Hmong volunteers. The mean coverage depths across the genome for each of the 11 samples ranged from 11 to 32×, with the mean being 22×, and 9,825,483 variant sites were identified against the reference genome (Fig. 1A). One donor, the relative of a blastomycosis patient, was flagged from further analysis because they did not have a confirmed history of blastomycosis. Of the 10 remaining donors with a history of confirmed blastomycosis, one additional donor was flagged because their risk for blastomycosis may have been modified by comorbidity (medical history of organ transplantation). Thus, WGS data from nine Hmong donors with a history of treatment for blastomycosis were used in the subsequent disease mapping strategy.

Operating under the hypothesis that susceptibility loci would be enriched in regions of autozygosity, we mapped runs of homozygosity (ROH). Consistent with other worldwide populations (10), the majority of ROH identified in Hmong cases are small (<49,277.2 bp using 3 clusters; see Fig. S1 in the supplemental material). We identified 27,514 consensus ROH (defined as regions where at least seven of the nine donors have overlapping ROH segments), which comprised 638,091,042 bp of the autosomes (Fig. 1A). The median consensus ROH was 8,773 bp in length, with the largest identified being 11,100,866 bp. ROH segments within 500 bp of another were merged prior to variant annotation and filtering (1,176 segments). We found 2,010,254 variants within these regions, of which 402,206 were fixed among all nine Hmong donors (Fig. 1A).

10.1128/mBio.01224-19.1 FIG S1 Characteristics of ROH identified in homozygosity mapping approach. ROH mapping algorithm comparison using 3 clusters; boundaries defining class A, B, and C ROH regions are bp 97801.9, 470624, and 25000000. On the *y* axis, the sum refers to the total number of base pairs in a given class, and *n* refers to the count of ROH in a given class. Download FIG S1, EPS file, 2.1 MB. Copyright © 2019 Merkhofer et al. 2019 Merkhofer et al. This content is distributed under the terms of the Creative Commons Attribution 4.0 International license.

These fixed single nucleotide polymorphisms (SNPs) were filtered based on frequencies of the alleles in European populations, as Hmong-Americans exhibit higher incidence rates of blastomycosis than do European-Americans (1). We imposed a threshold of 20%, resulting in 6,037 SNPs (Fig. 1A). We used three criteria to prioritize variants with a potential influence on fungal susceptibility (Fig. 1A; see also Materials and Methods) and identified 63 consensus ROH that harbor candidate susceptibility variants (Fig. 1B). Of 113 candidate susceptibility variants, 59 are annotated as regulatory by the Ensembl Variant Effector Predictor, 56 are within 200 kb of the transcription start site of an antifungal immunity gene, and 38 are demonstrated expression quantitative trait loci (eQTL) in the GTEx project (Fig. 1A to C and Table S1).

10.1128/mBio.01224-19.4 TABLE S1 Candidate susceptibility variants. Download Table S1, XLSX file, 0.1 MB. Copyright © 2019 Merkhofer et al. 2019 Merkhofer et al. This content is distributed under the terms of the Creative Commons Attribution 4.0 International license.

### *IL6* and *AS-IL6*, a long noncoding RNA, represent a candidate susceptibility locus.

**FIG 2:**
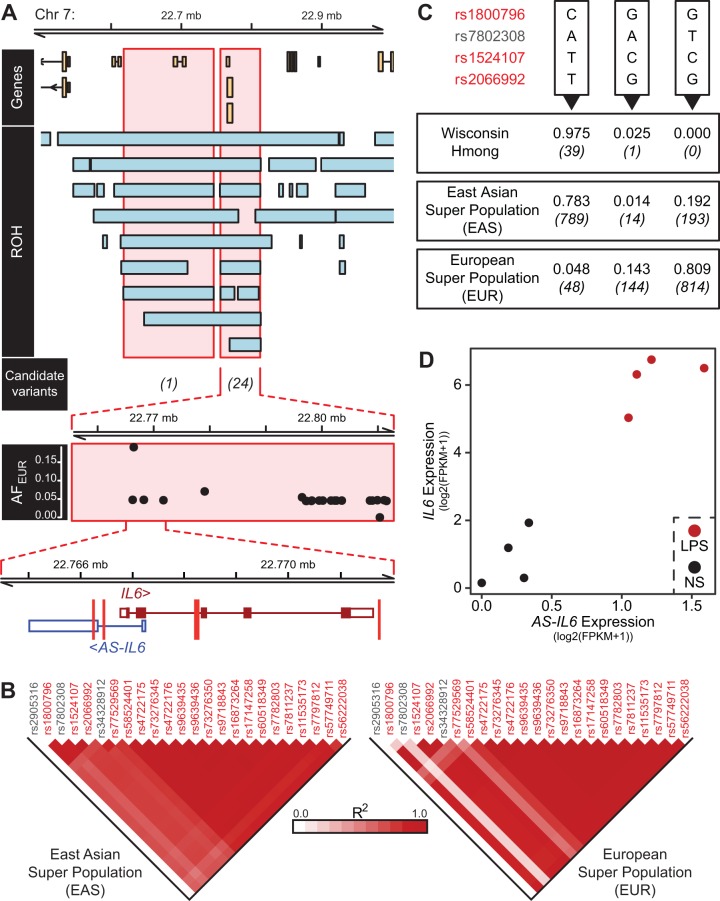
Candidate susceptibility variants at the *IL6*/*AS-IL6* locus are highly linked, representing a haplotype that is nearly fixed in WI Hmong. (A) Twenty-five candidate susceptibility variants located in two ROH that overlap the gene encoding IL-6 (*IL6*) and an antisense long noncoding RNA (lncRNA) (*AS-IL6*). This includes four SNPs that are either intronic or transcribed in *IL6* and *AS-IL6* (red vertical lines in bottom of panel A; two SNPs are 30 bp apart in *IL6* intron 2). (B) Linkage disequilibrium of candidate variants near *IL6*. Twenty-two of these variants, named in red text, are associated with *AS-IL6* expression. (C) SNPs closest to the *IL6* transcription start site were sequenced in a larger cohort of Hmong donors that also provided cells for B-LCLs (Fig. 3). CATT is the most common haplotype in Hmong individuals (AF_Hmong_, 0.975) and is common in East Asian populations (AF_EAS_, 0.783). CATT is rare in European populations (AF_EUR_, 0.048). (D) *AS-IL6* and *IL6* expression in monocytes left unstimulated (NS) or stimulated with LPS; reanalysis of RNA-sequencing data (EMTAB2399).

We manually inspected candidate variants and performed functional validation for one of the most promising loci. The gene encoding the cytokine IL-6 (*IL6*) is exceptional in our analysis in several ways. Our analysis revealed two consensus ROH near the locus on chromosome 7 between positions 22,617,358 bp and 22,812,578 bp containing 25 variants matching our filtering criteria (Fig. 1B and 2A). With 25 SNPs within 200 kb of the transcription start site, *IL6* is the gene associated with the most candidate susceptibility variants in our analysis (Table S1). The consensus ROH overlapping the *IL6* gene harbors 24 candidate susceptibility variants (Fig. 2A); the vast majority of consensus ROH do not harbor candidate variants, and no other consensus ROH harbors more than 4 candidate variants (median, 1 variant; Fig. 1D). Of note, 22 of the 25 SNPs identified near *IL6* are associated with expression of a long noncoding RNA (lncRNA) that overlaps *IL6* on the antisense strand (*AS-IL6*; AC073072.5 in the GTEx project). This likely reflects linkage disequilibrium between these SNPs (Fig. 2B). Nevertheless, *AS-IL6* is the gene associated with the most candidate susceptibility variants identified by our two unbiased criteria (Table S1). In addition to these features at the *IL6* locus, the encoded cytokine participates in the development of IL-17 responses, which are essential for host resistance in murine models of blastomycosis (9), lending biological plausibility to a role for the *IL6* locus in patients.

To further investigate the frequency of candidate SNPs at the *IL6* locus, we sequenced a subset of SNPs from a second cohort of Hmong donors (*n *=* *10) that did not report a history of blastomycosis. Though these SNPs are not fixed in the WI Hmong at large, they are at a higher frequency in the Hmong than in the East Asian population as a whole (allele frequency in Hmong [AF_Hmong_], 0.975; allele frequency in European ancestry [AF_EAS_], 0.783; Fig. 2C). This haplotype is rare in European populations (AF_EUR_ = 0.048; Fig. 2C).

*AS-IL6* is reported to play an important role in facilitating IL-6 production via H3K27ac in the *IL6* promoter (11). Intriguingly, one candidate SNP (rs1800796) is transcribed in *AS-IL6*, and an additional two candidate SNPs (rs1524107 and rs2066992) are upstream of *AS-IL6* in predicted transcription factor binding sites (SNP FuncPred, https://snpinfo.niehs.nih.gov/snpinfo/snpfunc.html). All three of these SNPs are reported eQTLs for *AS-IL6* but not for *IL6* in the GTEx project; a high degree of variability in *IL6* expression in postmortem tissues may interfere with the detection of eQTLs for this gene. Based on these data, we hypothesize that candidate susceptibility variants we have identified regulate *IL6* expression, possibly indirectly by impacting the regulatory lncRNA *AS-IL6*. In support of this hypothesis, we found a strong correlation between the *AS-IL6* expression and *IL6* when we reanalyzed published transcriptomic data from monocytes stimulated with lipopolysaccharide (LPS) (*R^2^* = 0.91, *P = *0.002) (12), which may reflect our hypothesis or a shared promoter (Fig. 2D).

### Functional validation of ethnic difference in cytokine production.

**FIG 3:**
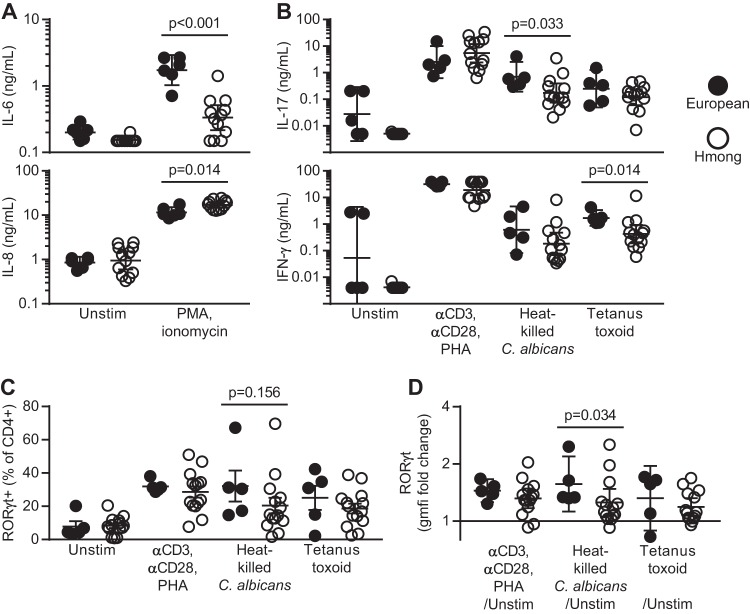
Stimulation of human B-LCL cells and primary memory CD4^+^ T cells reveals population differences in immune responses. (A) IL-6 and IL-8 production by B-lymphoblastoid cells (B-LCLs) from European and Hmong donors, which were grown synchronously and stimulated as shown or left untreated (Unstim). Each condition was run for each donor (*n_European_* = 6, *n_Hmong_* = 12) in technical triplicate, and two independent experiments were performed. Two donors (one European and one Hmong) are heterozygotes at SNPs of interest. Two other donors (both Hmong) have a history of confirmed blastomycosis. (B) IL-17 and IFN-γ production by memory CD4^+^ T cells, which were stimulated as shown or left untreated (Unstim). Each condition was run for each donor (*n_European_* = 5, *n_Hmong_* = 13) in technical quadruplicate, and all supernatants were analyzed simultaneously. (C and D) Percentage of cells that are RORγt^+^ (C), and expression of RORγt among activated memory CD4^+^ T cells (D) in cultures from panel B. Graphs display mean of technical replicates per donor and the geometric mean ± 95% confidence interval (CI) per group (panels A, B, and D) or mean ± standard error of the mean (SEM) per group (panel C). gmfi, geometric mean of fluorescence intensity.

While IL-17 responses have been implicated in murine models of B. dermatitidis infection, they have not been studied in humans with blastomycosis. Recognizing a unique opportunity to study the role of IL-6 and related IL-17 responses in human populations with known susceptibility, we quantified cytokine responses by Hmong and European donors’ cells. We were not able to reach Hmong blastomycosis patients for re-collection of primary cells; to circumvent this limitation, we created immortalized B-lymphoblastoid cells (B-LCLs) from Hmong and European subjects. B-LCLs from Hmong donors produced significantly less IL-6 than did B-LCLs from European donors in both the unstimulated (*P = *0.002) and stimulated (*P < *0.001) states (Fig. 3A). We also quantified IL-8 production as a control to see if there is a generalized difference in cytokine production; in contrast to IL-6, Hmong subject cells produced significantly more IL-8 upon stimulation than did European subject cells (Fig. 3A). Differences in IL-6 production remained significant (*P < *0.05) regardless of whether the two donors (one Hmong and one European) that are heterozygotic at SNPs near *IL6* were included or excluded from analysis. The difference in IL-8 production upon stimulation was similar between the two donor groups if the two heterozygotic donors are excluded (*P = *0.052). B-LCLs failed to produce cytokine when stimulated with fungal antigens (data not shown).

Our findings of an ethnic difference in IL-6 production, when correlated with genotype at the *IL6* locus, fit with past reports of human genetic differences in IL-6 production. Indeed, one candidate SNP (rs1800796) has been repeatedly associated with serum levels of IL-6 (13–16). These studies involve donors from different ethnicities and report contradicting relationships between genotype and IL-6 levels, perhaps indicating epistatic effects of these SNPs.

We also sought to measure antifungal immune responses in our donors downstream of IL-6. We surveyed memory CD4^+^ T cell responses to Candida albicans because it is likely that most healthy adults have been exposed to this fungus, and we hypothesized that deficits in IL-6 responses to fungi would affect this response. Hmong donor cells produced significantly less IL-17 than did European donor cells in response to C. albicans (*P = *0.033; Fig. 3B); Hmong donors also produced less IL-17 in the unstimulated state (*P = *0.012). Hmong donor cells likewise had lower expression of RAR-related orphan receptor gamma t (RORγt), the hallmark transcription factor of IL-17-producing T cells, in response to C. albicans than did European donor cells (Fig. 3C and D). The donor groups did not differ in IL-17 levels or RORγt expression in response to either direct T cell receptor (TCR) ligation (α-CD3, α-CD28, and phytohemagglutinin [PHA]) or the vaccine antigen tetanus toxoid (Fig. 3B to D). These data suggest that impaired IL-6 production in the Hmong subjects may affect IL-17-producing T cells in response to fungi more generally.

We did not detect differences in IFN-γ responses to heat-killed *Candida* or TCR ligation but did find that cells from Hmong donors produced less gamma interferon (IFN-γ) than did European donors in response to tetanus toxoid (Fig. 3B). Our genomic analysis of Hmong blastomycosis patients leads us to hypothesize that these IL-17 differences by memory CD4^+^ T cells in response to fungal antigen are downstream of IL-6. To investigate whether ethnic differences in innate (myeloid) IL-6 or adaptive (IL-6 dependent) IL-17-producing T cell responses would be more likely to account for patient susceptibility to blastomycosis, we studied the loss of IL-6 signaling in murine models of innate and adaptive immunity to B. dermatitidis.

### IL-6 is required for the development of protective T cells and neutrophil responses in a murine model of blastomycosis.

IL-6 promotes the development of T helper 17 (Th17) responses (17), and murine models of antifungal immunity have demonstrated that Th17 cells foster acquired resistance to experimental pulmonary blastomycosis (9). We investigated the contribution(s) of IL-6 to innate and acquired resistance in the murine model of blastomycosis.

**FIG 4:**
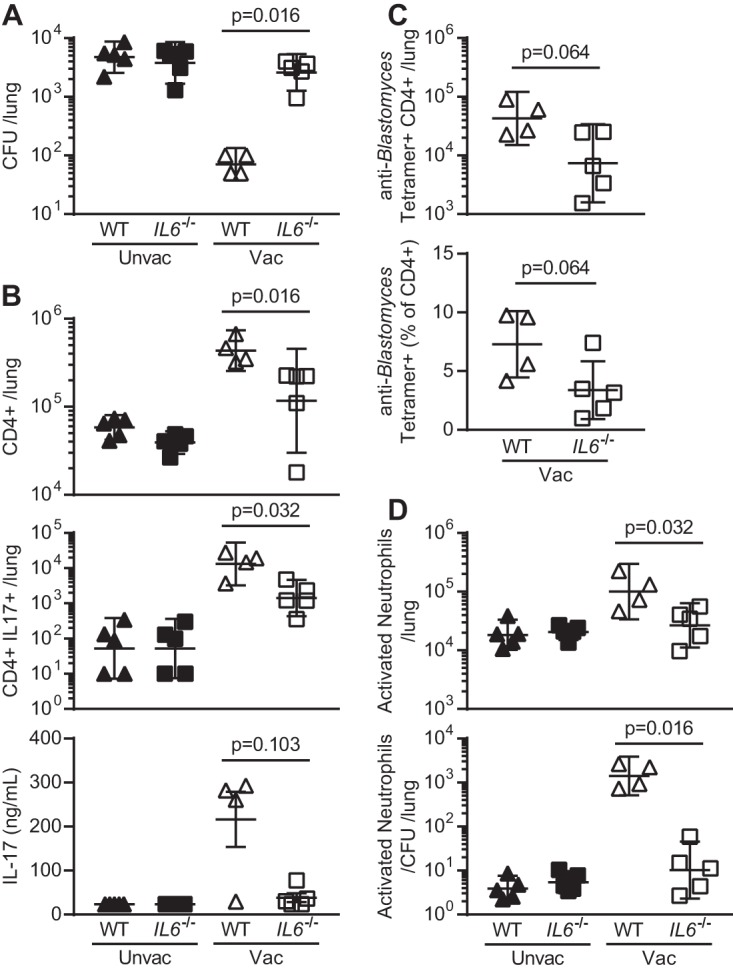
IL-6 is required for the development of acquired resistance in a mouse model of blastomycosis. Mice were vaccinated (Vac) or not (Unvac) and challenged with WT B. dermatitidis (*n *=* *4 to 5 males/group). (A) Lung CFU 4 days postinfection. (B) Numbers of CD4^+^ T cells and IL-17^+^ CD4^+^ T cells recruited to lung 4 days postchallenge, and IL-17 production by splenocytes in response to *ex vivo* stimulation with B. dermatitidis antigen. (C) Number and percentage of tetramer-positive *Blastomyces*-specific CD4^+^ T cells. (D) Activated neutrophils in lung, both by absolute number and relative to CFU. No significant difference was found between unvaccinated WT and *IL6*^−/−^ groups among any parameter depicted. Graphs display individual mice and geometric mean ± 95% CI per group. Data are representative of two experiments performed, although tetramer staining in panel C was done on 4 to 5 mice from one experiment.

We found that IL-6 had a requisite role in the development of adaptive immunity and resistance to B. dermatitidis. In a vaccine model, *IL6*^−/−^ mice failed to acquire resistance to lethal experimental challenge, as evidenced by significantly higher fungal burden than in wild-type (WT) mice (Fig. 4A). *IL6*^−/−^ mice recruited significantly fewer total CD4^+^ T cells and IL-17-producing CD4^+^ T cells to the lung upon fungal challenge than did vaccinated WT mice (Fig. 4B). IFN-γ responses were similar between the groups (Fig. S2A). Splenocytes from the *IL6*^−/−^ mice likewise revealed a trend toward less IL-17 than those from WT mice upon *ex vivo* stimulation with B. dermatitidis antigen (Fig. 4B). Upon excluding one outlier in the WT vaccinated group (Grubbs’ test, *P < *0.05), this difference in IL-17 production between groups reached significance (*P = *0.036). There was also a trend toward vaccinated *IL6*^−/−^ mice having fewer tetramer-positive *Blastomyces*-specific CD4^+^ T cells and these cells representing a smaller portion of CD4^+^ T cells in the lungs of *IL6*^−/−^ mice than their WT counterparts (Fig. 4C).

10.1128/mBio.01224-19.2 FIG S2 Features of antifungal immunity in mice. (A) IFN-γ^+^ CD4^+^ T cells and IFN-γ production by murine splenocytes in response to *Blastomyces* antigen. (B) Total neutrophil recruitment upon infection and corrected for fungal burden. (C) CFU following B. dermatitidis infection of naive WT mice in groups that received IL-6-neutralizing antibody (α–IL-6) or isotype control (Iso) (*n *=* *10 females/group). Graphs display individual mice and the geometric mean ± 95% CI per group. Download FIG S2, EPS file, 1.9 MB. Copyright © 2019 Merkhofer et al. 2019 Merkhofer et al. This content is distributed under the terms of the Creative Commons Attribution 4.0 International license.

Past reports have sought to characterize cellular sources of IL-17 in blastomycosis and have implicated, in addition to Th17 cells, Tc17 cells, γδ T cells, natural Th17 (nTh17) cells, and innate lymphoid cells (18, 19). In fact, previous reports have demonstrated that the loss of IL-6 leads to a decrease in Tc17 responses to Blastomyces spp., though this was carried out in CD4^+^ T cell-deficient mice (19). Though outside the scope of this study, it is possible that other lymphocyte populations in addition to Th17 cells contribute to the IL-17 phenotype we report.

Neutrophils are essential in resistance to B. dermatitidis (20). Compared to vaccinated WT mice, *IL6*^−/−^ mice evinced a significant paucity of activated neutrophils in the lung, as well as total neutrophils, especially when corrected for fungal burden (Fig. 4D and S2B). In contrast to the model of acquired resistance, antibody neutralization of IL-6 in unvaccinated WT mice failed to influence resistance to infection (Fig. S2C). Likewise, unvaccinated WT and *IL6^−/−^* mice did not differ on any endpoint analyzed (Fig. 4A to D). Taken together, our findings suggest that IL-6 plays a more prominent role in acquired versus innate resistance to this infection.

Our findings demonstrate that IL-6 is critical to adaptive immunity to B. dermatitidis infection. Considering that IL-6 promotes the development of IL-17-producing CD4^+^ T cells (17), our findings in both the murine model and the human donors are consistent with past work that identified a role for IL-17 in acquired resistance to experimental blastomycosis in mice (9). While we could not compare memory CD4^+^ T cell responses to *Blastomyces* spp. in Hmong and European patients, our use of C. albicans as a surrogate stimulus raises the possibility that lower IL-6 production in the WI Hmong relative to other populations may have broader implications for antifungal immunity.

In summary, we characterize genetic differences that may underlie susceptibility to an endemic mycosis and experimentally analyze population differences in antifungal immunity. Analysis of Hmong blastomycosis patient genomes revealed 113 candidate susceptibility variants. We experimentally validated the *IL6* locus, which harbors 25 of the candidate variants, more than any other antifungal immunity gene in our analysis. Cells from Hmong donors produced less IL-6 than did cells from European donors and blunted IL-17 production by memory CD4^+^ T cells in response to fungi. We also formally demonstrated that the loss of IL-6 engenders susceptibility and blunts IL-17-producing CD4^+^ T cells in a murine model of acquired resistance to B. dermatitidis.

Our data in humans and mice support our contention that genetic variation at the *IL6* locus, and altered IL-6 responses to fungi, contribute to susceptibility to fungal infection. Loss of IL-6 signaling in humans confers a risk of pneumonia, as evinced by long-term follow-up of patients treated with IL-6 receptor (IL-6R) blockade (21). IL-6 promotes Th17 responses, and a number of genetic defects affecting Th17 have been reported to alter susceptibility to other fungal pathogens, including other endemic mycoses (4) and C. albicans (22). Th17 responses also play a role in the control of other intracellular pathogens, including Mycobacterium tuberculosis (23, 24). Intriguingly, the genotype at one candidate susceptibility variant that we report near *IL6* is correlated with susceptibility to tuberculosis and hepatitis B virus in Han Chinese populations, with the proposed mechanisms involving the development of Th17 responses (16, 25). Clinical recommendations regarding patients on immunologic blockade with biological agents already recommend screening and prophylaxis for granulomatous diseases, including tuberculosis and histoplasmosis, though this is best characterized in the context of tumor necrosis factor alpha (TNF-α) blockade (26). It may be prudent for clinicians managing these patients to also have higher suspicion of blastomycosis.

A number of highly linked candidate variants at the *IL6* locus are eQTLs for *AS-IL6*, a long noncoding RNA that was recently reported to be involved in IL-6 production following stimulation (11). Indeed, one candidate variant is transcribed in *AS-IL6*. We hypothesize that candidate variant(s) may engender susceptibility to blastomycosis in humans by altering the regulation of *IL6*, possibly by way of quantitatively or qualitatively altering *AS-IL6*. Our future experiments will be directed at characterizing the role of select SNPs on the IL-6/*AS-IL6* system.

Although we were constrained by the fact that blastomycosis is a relatively uncommon infection, we circumvented this problem by implementing an innovative ROH mapping strategy to investigate the genetic basis of a rare condition and implicate a potential susceptibility locus.

We acknowledge that the size of our study is small and that identification of candidate susceptibility variants was biased toward 108 genes postulated or implicated in immunity to fungi. However, we also created additional criteria for prioritization, specifically, polymorphisms flagged as regulatory, and those that are reported eQTLs. Of 113 candidate variants we report here, only 26 were identified solely on the basis of their proximity to potential antifungal immunity genes. Future studies will seek to apply unbiased informatics approaches, including studies with animal models that are not feasible to carry out with human donors when studying a rare disease. These studies may uncover numerous susceptibility loci not previously implicated in antifungal immunity, which will enable the discovery of polygenic susceptibility variants. We are optimistic that such studies will facilitate future human genetic studies, including broader analysis of genetic susceptibility to blastomycosis among the Hmong people.

## MATERIALS AND METHODS

### Ethics statement.

All human subject research was approved by the institutional review board of the University of Wisconsin—Madison (project ID 2013-1139). Studies involving laboratory animals were approved by the IACUC of UW-Madison (project ID M005891).

### Whole-genome sequencing.

After obtaining informed consent, we collected saliva samples from individuals of Hmong ancestry. All but one donor had a history of treatment for confirmed blastomycosis, including five individuals who presented with blastomycosis associated with an outbreak in 2013 in Marathon County, WI (1). Illumina sequencing was performed at the University of Wisconsin Biotechnology Center or the Broad Institute. Variant calls for both sets of sequencing runs were done by the Broad Institute using their established best practices pipeline for quality control and mapping of human sequencing data. The GRCh37/hg19 build was used as the human genome reference.

### Runs of homozygosity.

Prior to identification of ROH, variants were filtered with VCFtools using the following parameters: –max-missing 0.5, –minQ 30, –minDP 3, –remove-indels, –max-alleles 2, –hwe 0.0001. BCFtools/RoH (27) and GARLIC (28) were used to identify ROH in each study participant. Allele frequencies were calculated within GARLIC by resampling 50 individuals from nine unrelated Hmong individuals using the –freq-only flag. The parameters used to calculate ROH with Garlic were garlic –build hg19 –error 0.001 –auto-winsize –auto-overlap-frac –winsize 50; the –freq-file generated from the unrelated individuals was provided; and default clusters as well as a run with –nclust 5 were run. The same frequencies were provided to the BCFtools/RoH algorithm: bcftools roh –G30 –AF-file. Visualization of ROH identified by both programs was performed with ggplot2 in R (https://cran.r-project.org/web/packages/ggplot2/index.html). Recognizing imprecision in identifying ROH, we merged individual ROH calls from the two algorithms using bedtools merge. We looked for overlapping ROH among cases using BEDOPS (29) and focused on regions in which at least seven of the nine cases had an ROH identified; we refer to these regions as “consensus ROH.”

### Candidate susceptibility variants.

Variants within consensus ROH that were fixed in Hmong blastomycosis cases and found at <20% frequency in the European (EUR) Superpopulation of the 1000 Genomes Project (30) were inspected to identify candidate susceptibility variants. We focused on variants that fulfilled at least one of three criteria, (i) proximity to antifungal immunity genes, (ii) annotation as regulatory, or (iii) reported expression quantitative trait loci (eQTL).

Starting from a list of genes in an antifungal immunity array and adding genes based on the literature, we interrogated 108 genes known or suspected to be involved in human immune responses to fungi (Table S2). We flagged SNPs occurring within 100 kb on either side of the transcriptional start site of these genes. SNPs within consensus ROH were annotated using the Ensembl Variant Effector Predictor (31) and cross-referenced with the 1000 Genomes and Genotype-Tissue Expression (GTEx; https://gtexportal.org/home/) projects to identify variants with predicted or demonstrated effects on function.

10.1128/mBio.01224-19.5 TABLE S2 Antifungal immunity genes. Download Table S2, XLSX file, 0.1 MB. Copyright © 2019 Merkhofer et al. 2019 Merkhofer et al. This content is distributed under the terms of the Creative Commons Attribution 4.0 International license.

### RNA-sequencing analysis.

RNA-sequencing reads (FASTQs) for 8 samples from a previous study (12), accession number EMTAB2399, were mapped to the human genome (hg19) with the STAR aligner v.2.5.0a (32). We used Qualimap ‘bamqc’ and ‘rnaseq’ for quality control assessment (33, 34). StringTie v.1.3.3 and Ballgown (35) were used to identify and quantify expression levels in fragments per kilobase of transcript per million mapped reads (FPKM) for each annotated transcript (GENCODE release 27). Gene expression matrices were correcting for GC percentage and 5′→3′ bias.

### B-Lymphoblastoid cells.

Donors were selected by ancestry (self-reported), and to the best of our knowledge, no donors are first-degree relatives. Peripheral blood mononuclear cells (PBMCs) were isolated from blood of donors using Ficoll-Paque Plus (GE Healthcare). B-LCLs were generated by treating PBMCs with Epstein-Barr virus and then maintained in Iscove’s modified Dulbecco’s medium supplemented with 16% fetal bovine serum. Cultures tested negative for Mycoplasma spp. prior to use in cytokine assays (MycoProbe; R&D Systems).

### Donor genotype at *IL6* locus.

Genomic DNA was isolated from B-LCLs, and the *IL6* locus was amplified by PCR. The product was gel purified and ethanol precipitated. Sanger sequencing was performed. MacVector 15.1.3 was used to align sequences. Population frequencies and linkage between SNPs were calculated from the 1000 Genomes Project using LDlink (https://ldlink.nci.nih.gov/).

### B-LCL cytokine production.

B-LCLs were grown synchronously and plated (1 × 10^6^ cells/well) in 24-well plates. Wells were left unstimulated or stimulated (phorbol 12-myristate 13-acetate [PMA; 50 ng/ml] and ionomycin [250 ng/ml]). Supernatant was harvested after 24 h, and IL-6 and IL-8 (control) contents were measured by enzyme-linked immunosorbent assay (ELISA; ELISA Max kits; BioLegend). The data were analyzed either based on ancestry alone (including all donors) or including only donors who were homozygotic at 3 SNPs of interest (rs1800796, rs1524107, and rs2066992) following *a priori* expectations based on ancestry.

### Human CD4^+^ T cell stimulation.

Studies of immunologic memory were patterned after previously reported studies of primary CD4^+^ T cell responses to C. albicans, among other stimuli (36). Magnetic beads were used to isolate monocytes (CD14^+^) and memory CD4^+^ T cells (CD45RO^+^) from donor PBMCs (CD14 MicroBeads, Miltenyi; and MojoSort human memory CD4^+^ T cell isolation kit; BioLegend). Cells were plated (3 × 10^5^/well) in 24-well plates and maintained in RPMI supplemented with 10% autologous serum, sodium pyruvate, nonessential amino acids, and glutamine. Treatments included a ratio of 1:1 CD14^+^ and CD45RO^+^ cells, except the positive control, which included only CD45RO^+^ cells. Stimuli were medium alone, positive control (α-human CD3 antibody coated on plate [2 μg/ml], α-human CD28 antibody [2 μg/ml], and PHA [5 μg/ml]), heat-killed C. albicans (1.2 × 10^6^ yeast/well), or tetanus toxoid (3 μg/ml). After 4.5 days, supernatant was harvested and stored at −20°C, and cells were analyzed by flow cytometry. IL-17A and IFN-γ were quantified in supernatants in one run by a Luminex assay (R&D Systems).

### Fungi.

The attenuated B. dermatitidis strain 55 that lacks *BAD1* and the isogenic wild-type (WT) virulent strain ATCC 26199 were used (37). These yeasts were grown at 39°C on Middlebrook 7H10 agar with an oleic acid-albumin complex. C. albicans strain K1 was grown on Sabouraud (SAB) dextrose agar. Candida spp. and B. dermatitidis strain 55 were killed by heating to 65°C for 30 min.

### Mice and infection.

Wild-type C57BL/6J mice were bred under specific-pathogen-free conditions at UW-Madison. *IL6*^−/−^ mice (B6.129S6-Il6^tm1Kopf^) were obtained from Jackson Laboratory. All mice were age matched. Mice were vaccinated twice, subcutaneously, 2 weeks apart with 2 × 10^6^ heat-killed B. dermatitidis strain 55. Two weeks after the boost, mice were infected intratracheally with 2 × 10^4^ ATCC 26199 virulent yeasts. In some experiments, naive mice received 500 μg anti-IL-6-neutralizing antibody (MP5-20F3; Bio X Cell) or isotype control on the day of infection and 2 days later. Lungs were harvested 4 days postinfection and cells analyzed by flow cytometry; homogenates were plated on brain heart infusion (BHI) agar and colonies counted 7 days later.

### Mouse splenocyte stimulation.

Spleens were manually disrupted and splenocytes plated in 96-well plates (1 × 10^6^ cells/well). Cells were left unstimulated or stimulated with cell wall membrane antigen of B. dermatitidis (37). After 3 days, culture supernatants were harvested, and IL-17A and IFN-γ contents were measured by ELISA (R&D Systems).

### Flow cytometry.

Human cells were stained for surface CD4, CD44, and CD45RO and intracellular RORγt (Fig. S3A). Dead cells were labeled with LIVE/DEAD fixable near-infrared (near-IR) (Invitrogen). Surface markers were stained on ice for up to 60 min. Intracellular markers were analyzed using the eBioscience Foxp3/transcription factor staining buffer set. After cell permeabilization at 4°C for at least 60 min, intracellular markers were stained at 4°C overnight.

10.1128/mBio.01224-19.3 FIG S3 Gating strategy. (A) Gating strategy for identification of human memory T cells (CD4^+^ CD44^+^ CD45RO^+^) and RORγt^+^ cells. Gating of CD44 and CD45RO is based on fluorescence-minus-one controls. (B) Gating strategy for identification of murine CD4^+^ T cells, including intracellular stains. (C) Gating strategy for identification of murine total (CD11b^hi^ Ly6G^+^) and activated (CD11b^hi^ Ly6G^+^ Ly6C^hi^) neutrophils. Download FIG S3, PDF file, 0.3 MB. Copyright © 2019 Merkhofer et al. 2019 Merkhofer et al. This content is distributed under the terms of the Creative Commons Attribution 4.0 International license.

Mouse lungs were mechanically disrupted (GentleMACS dissociator; Miltenyi), enzymatically digested (2 mg/ml collagenase D and 10 μg/ml DNase, 37°C, 20 min), and disrupted a second time. Cells were separated by Percoll gradient. Mouse cells were stained for surface CD4, CD8, CD11b, CD11c, CD44, CD90.2, NK1.1, B220, *Blastomyces* endoglucanase 2 (Bl-Eng2)-specific tetramer (H. Dobson, L. Dias, L. Kohn, S. Fites, D. Wiesner, T. Dileepan, G. Kujoth, G. Ostroff, B. Klein, M. Wüthrich, unpublished data), and intracellular IFN-γ and IL-17A (Fig. S3B and C). Dead cells were labeled with LIVE/DEAD fixable near-IR (Invitrogen). Surface markers were stained on ice for up to 60 min. Intracellular markers were analyzed using BD Cytofix/Cytoperm. After cell permeabilization at 4°C overnight, intracellular markers were stained on ice for up to 60 min.

Count beads (UltraComp eBeads; Invitrogen) were included with each sample to calculate absolute cell numbers. All samples were run through a five-laser BD LSRFortessa, and data were analyzed with the FlowJo software v.10.

### Statistical analysis.

Data were analyzed in GraphPad Prism 7.04 using the two-tailed Mann-Whitney test. A *P* value of ≤0.05 was defined as statistically significant.
