# Supplementary figures and images for: Investigation of Genetic Susceptibility to Blastomycosis Reveals Interleukin-6 as a Potential Susceptibility Locus
Source: mBio. 2019 Jun 18;10(3):e01224-19. doi: 10.1128/mBio.01224-19 (PMC6581865; doi:10.1128/mBio.01224-19)

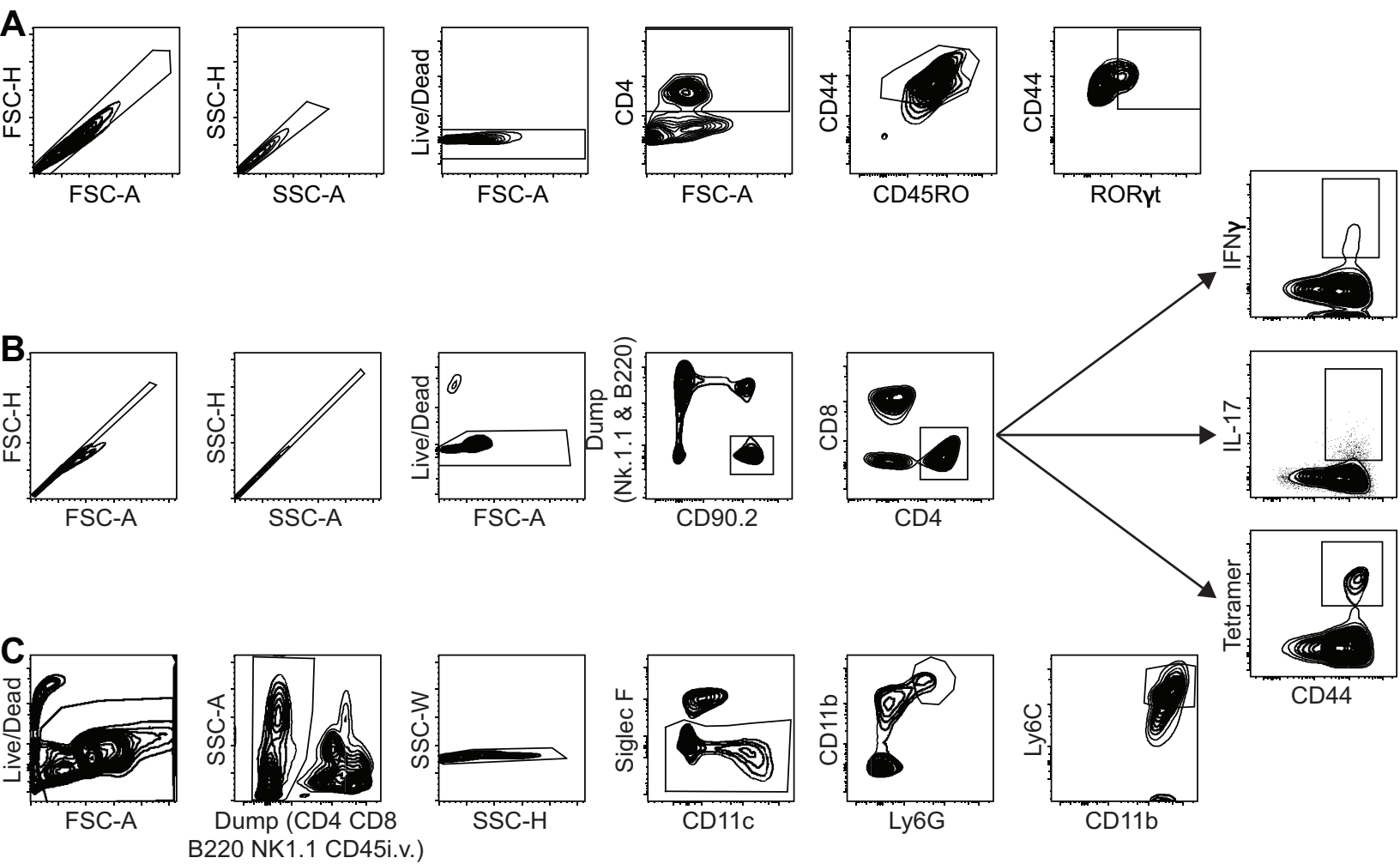

Supplement: FIG S3 [file mBio.01224-19-sf003.pdf]
